# Effects of Industrial Structure Adjustment on Pollutants Discharged to the Aquatic Environment in Northwest China

**DOI:** 10.3390/ijerph19106146

**Published:** 2022-05-18

**Authors:** Chenyu Lu, Xianglong Tang, Wei Liu, Ping Huang

**Affiliations:** 1 School of Architecture and Urban Planning, Lanzhou Jiaotong University, Lanzhou 730070, China; tangxl@mail.lzjtu.cn; 2 Yulin Huadong Middle School, Yulin 719000, China; lw1727175427lw@163.com; 3 College of Geography and Environmental Science, Northwest Normal University, Lanzhou 730070, China; hp18379536235@163.com

**Keywords:** aquatic environment, industrial structure, vector autoregressive, impulse response function, Northwest China

## Abstract

Northwest China is located along China’s Belt and Road Initiative routes and represents the frontier and core region for China’s construction and development of the Silk Road Economic Belt. In recent years, the conflict between economic development and environmental pollution has become increasingly intense in this region, with the latter mainly caused by disorderly industrialization brought about by rapid urbanization processes. Inappropriate industrial structure is the primary reason for environmental degradation in Northwest China, which has limited precipitation and available water. Due to its fragile aquatic environment and unsustainable use of water resources, the pollution and degradation of the aquatic environment has become a bottleneck that severely restricts the sustainable development of China’s northwest region. In the present study, five provinces or autonomous regions in Northwest China were selected as the study objects. Based on the vector autoregressive (VAR) model, quantitative research methods, such as impulse response function and variance decomposition analysis, were applied to quantify the dynamics between industrial structure adjustment and changes in industrial pollutant discharges to the aquatic environment, so that the impact of industrial structure adjustment on pollutants discharged to the aquatic environment could be quantified and characterized. Therefore, the present study has both theoretical and practical significance. The conclusions are as follows: (1) In general, industrial structure in most provinces in Northwest China imposes a positive effect over the discharge of pollutants to the aquatic environment. Adjusting industrial structure and reducing the proportion of secondary industry present can to some extent promote reductions in the discharge of pollutants to the aquatic environment. However, such beneficial effects may vary among different provinces. (2) Specifically, for Gansu, province industrial structure adjustment could help reduce the discharge of pollutants to the aquatic environment effectively during the early stages, but this positive effect gradually weakens and disappears during the later stages. In Qinghai province, industrial structure adjustment could not help reduce the discharge of pollutants to the aquatic environment effectively during the early stages, but a positive effect gradually increases and continues to function later. The performance in Shaanxi and Xinjiang provinces was quite similar, with industrial structure adjustment helping to effectively reduce the discharge of pollutants to the aquatic environment over a long period of time. This positive effect can play a more sustained and stable role. For Ningxia province, industrial structure adjustment can not only help significantly reduce the discharge of pollutants to the aquatic environment but also displays a significant positive effect. (3) Given the specific conditions and characteristics of the region under study, relevant policies for industrial structure adjustment should be formulated and implemented.

## 1. Introduction

Climate change, environmental degradation, and other challenges have become the key agenda of global governance, and they also represent the core elements affecting the economy, society, security, stability, and future development of regions along China’s Belt and Road Initiative (BRI) routes [1]. The United Nations has repeatedly emphasized climate emergency issues and called for international cooperation to deal with overlapping global crises, such as climate change, biodiversity loss, and environmental pollution [2]. In 2017, China’s Ministry of Ecology and Environment, Ministry of Foreign Affairs, Ministry of Commerce, and National Development and Reform Commission jointly issued “Guidance on Promoting Green Belt and Road”, formally proposed the promotion of “green” belt and road routes, and clarified the reasons for promoting the BRI and its essence [3]. With the promotion of the BRI, China is striving to achieve high-quality development, with the development of five provinces or autonomous regions in Northwest China as one of the top priorities [4].

**Figure 1:**
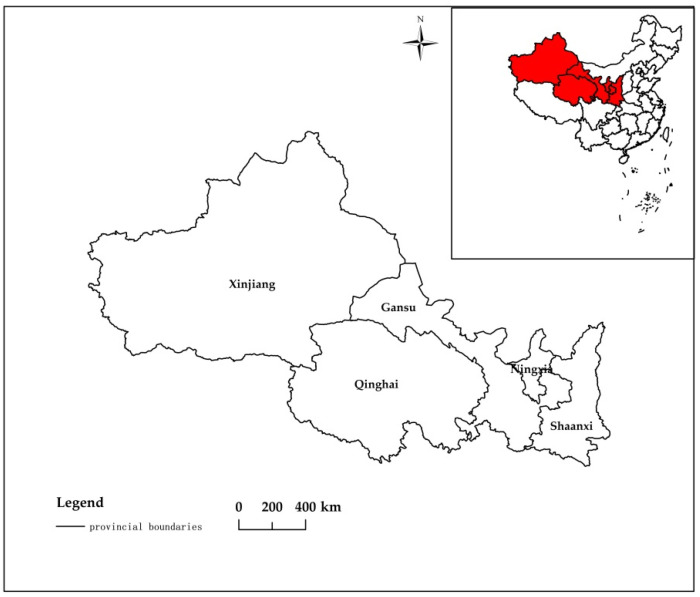
Schematic diagram of the specific locations of the study objects.

Northwest China is located along the belt and road routes and represents the frontier and core region for China’s construction and development of the Silk Road Economic Belt, which includes five provincial-level administrative regions: Shaanxi, Gansu, Qinghai, Ningxia Hui Autonomous Region, and Xinjiang Uygur Autonomous Region (Figure 1). In recent years, the conflict between economic development and environmental pollution has become increasingly intense in this region, with the latter mainly caused by disorderly industrialization brought about by rapid urbanization processes [5,6,7,8]. Industrial structure, the degree of urbanization, scientific and technological development, and population size are all important factors that affect the extent of environmental pollution [9,10,11,12]. Inappropriate industrial structure is the primary reason for environmental degradation in Northwest China, which has limited precipitation and available water. Due to its fragile aquatic environment and unsustainable use of water resources, the pollution and degradation of the aquatic environment has become a bottleneck that severely restricts the sustainable development of China’s northwest region [13]. Industrial structure is an important link that connects anthropogenic activities and the ecological environment in our modern society. It not only functions as a resource configurator but also serves as a control body that determines the rate at which environmental resources are consumed, as well as the quality and quantity of pollutants generated [14]. Given these facts, there is an urgent need to conduct research to analyze the impact of industrial structure adjustment on the discharge of pollutants to the aquatic environment in Northwest China and to seek a coordinated path toward sustainable development that balances economic growth with environmental protection.

In the present study, five provinces or autonomous regions in Northwest China were selected as the study objects. Based on the vector autoregressive (VAR) model, quantitative research methods, such as impulse response function and variance decomposition analysis, were applied to quantify the dynamics between industrial structure adjustment and changes in industrial pollutant discharges in the aquatic environment, so that the impact of industrial structure adjustment on pollutants discharged to the aquatic environment could be quantified and characterized. On the one hand, the present study can help supplement and improve current research into environmental economics, both theoretically and empirically, and enrich the theoretical content of human geography and sustainable development, i.e., it has theoretical significance. On the other hand, this study lays a scientific foundation for further optimization of the industrial structure and the promotion of environmental protection in Northwest China, providing theoretical support and a decision-making basis for promoting the construction of ecological civilization and the implementation of sustainable development strategies. Therefore, it also possesses important practical significance.

## 2. Literature Review

To date, relevant international studies based on the perspective of industrial structure and environmental pollution have mainly focused on two aspects, namely the analysis of the relationship between industrial structure and environmental pollution and how to reduce the discharge of pollutants through industrial structure adjustment. Linear or nonlinear relationships can be used to describe the relationships between industrial structure and environmental pollution, with nonlinear relationships being the most common. In terms of linear relationships, Cole et al. showed that there was a linear relationship between industrial structure and environmental pollution [15]. In terms of nonlinear relationships, studies by Grossman [16], Lee [17], and Chen [18] all showed that there was an inverted U-shaped relationship between industrial structure and environmental pollution. However, it is worth noting that the environmental Kuznets curve (EKC) relationship depends to a large extent on the nature of the pollution indicators and estimation methods used; the mechanisms between different pollutants and the industrial structures involved might be completely different and display a variety of relationships, such as a U-shaped relationship [19], an inverted-N-type relationship [19], or an N-type relationship [20]. Victor [21] and Jalil [22] reported that there was no inverted U-shaped relationship between industrial structure and environmental pollution, and they suggested that technological progress and innovation have played important roles in reducing the discharge of pollutants to the environment. As for studies that have aimed to reduce the discharge of pollutants to the environment through industrial structure adjustment, Mougou et al. used the impulse response method to conduct an analysis and proposed that various industries in Tunisia should formulate different pollutant reduction policies [23]. Cherniwchan et al. applied the neoclassical growth model and found that a reduction in the input share by industrial sectors led to a corresponding reduction in the discharge of pollutants [24]. Jalil et al. found that a reduction in the proportion of energy-intensive industries could help reduce the discharge of pollutants [22].

In China, related research based on the perspective of industrial structure and environmental pollution is generally consistent with global research trends and has also mainly focused on the two aspects mentioned above. Research into the patterns between industrial structure and environmental pollution has similarly focused on linear or nonlinear relationships, with nonlinear relationships being the most common pattern explored. In terms of linear relationships, Wang et al. found a linear relationship between industrial structure and environmental pollution [25]. In terms of nonlinear relationships, Xu et al. reported a positive N-type relationship between environmental pollution and industrial structure in Jiangxi [26]. Li et al. reported that there was an inverted U-shaped relationship between China’s industrial structure and environmental pollution [27]. Zhang et al. [28] and Niu et al. [29] carried out research into the nonlinear relationship between industrial structure and pollution of the aquatic environment using different methods and mixing a variety of perspectives. With regard to studies that aimed to reduce the discharge of pollutants to the environment through industrial structure adjustment, Meng et al. suggested that a reasonable adjustment of industrial structure is beneficial for handling resource and environmental constraints [30]. Sun [31], Zhang [32], and other researchers used panel data models to demonstrate that industrial structure adjustment could help reduce the discharge of pollutants. Han et al. applied quantitative spatial models and showed that industrial structure adjustment is beneficial for the improvement of ecological efficiency [33].

Overall, the relevant research conducted thus far has achieved considerable fruitful results, although some shortcomings still exist. First, quantitative research that tests the impact of industrial structure adjustment on the discharge of pollutants to the aquatic environment has yielded limited results and is still in the preliminary stages. Second, based on vector autoregression (VAR) models, researchers have systematically applied quantitative methods, such as impulse response function and variance decomposition, and provided a comprehensive quantitative analysis of the dynamics between industrial structure adjustment and changes in the quantity of pollutants discharged to the aquatic environment. However, these research accomplishments have been insufficient, and further research is urgently needed. Third, provincial-level systematic and comprehensive comparative research that covers the entire northwestern region of China is relatively lacking. In sum, the present study could help to address the above-mentioned shortcomings and deficiencies.

## 3. Data and Methods

### 3.1. Indicators and Data

For the present study, we selected two indicators that characterize and reflect changes in industrial structure and the discharge of pollutants to the aquatic environment. In terms of changes in industrial structure, we have consulted existing research [34,35,36] and selected the proportion of secondary industry (SI) as the representative indicator, which is the proportion of the added value of secondary industry to regional gross domestic product (GDP). Two of the main indicators commonly used to characterize water quality, chemical oxygen demand (COD) and ammonia nitrogen (AN), have been closely monitored in China since the implementation of the eighth Five Year Plan; these indicators are widely used in the assessment of water quality as they are considered to be both typical and representative [37,38]. Therefore, in the present study, COD and AN in industrial wastewater were selected as representative indicators that reflect the extent of the discharge of pollutants to the aquatic environment. This includes two aspects: the direct discharge of industrial wastewater and the aerial discharge that is deposited on land and subsequently washed into water courses via rainfall.

The present study took 2001 to 2019 as the research period, with all original data taken from the China Statistical Yearbook and the China Environmental Yearbook series. The natural logarithmic transformation of data does not change its original cointegration relationship and maintains the original characteristics of the data. However, this transformation helps to linearize data and eliminate the heteroscedasticity of time series, thus providing stationary series that are more convenient for further analyses [39,40]. Therefore, logarithmic processing was performed on the initial time-series data of all obtained indicators, which are represented by lnSI, lnCOD, and lnAN.

### 3.2. Research Methods

#### 3.2.1. The Vector Autoregression Model

The VAR model, first proposed in 1980 by Christopher A. Sims, is often used to capture the relationship between multiple quantities as they change over time and to characterize the dynamic effects of random perturbation terms on variable systems [41]. In general, a VAR(*p*) model with k variables can be expressed as follows:

(1) $$y_{t}=A_{1}y_{t-1}+A_{2}y_{t-2}+\cdots +A_{p}y_{t-p}+B_{1}x_{t}+\cdots +B_{r}x_{t-r}+\varepsilon_{t}\left(t=1,2,\cdots ,T\right)$$

In Equation (1), $y_{t}$ represents a *k*-dimensional endogenous variable, $x_{t}$ represents an *f*-dimensional exogenous variable, and the sample size is *T*. A1,A2,⋯AP and B1,B2,⋯Br represent the parameter matrix to be estimated, p and r represent the lag coefficients of endogenous variables and exogenous variables, respectively, and $\varepsilon_{t}$ represents k-dimensional vectors with random perturbations.

#### 3.2.2. Impulse Response Function and Variance Decomposition

Both a stationarity test and a cointegrating relationship test are required prior to any analysis of the impulse response function [42,43]. In the present study we applied the augmented Dickey–Fuller test to test the stationarity of each variable in the time series, based on the logarithmic processing of the time-series data. For the cointegration test, we evaluated the cointegrating relationships among multiple variables, and the Johansen cointegration test was specifically performed to test whether long-term equilibrium relationships exist among variables.

The impulse response function is used to quantitatively analyze the long-term dynamic relationships between changes in industrial structure and the discharge of pollutants to the aquatic environment, according to the following formula [44]:

(2) $$y_{t}=c^{0}\varepsilon_{t}+c^{1}\varepsilon_{t-1}+c^{2}\varepsilon_{t-2}$$

In this formula, the response function due to the impulse of *y_i_* is $c_{ij}^{0}$*,*$c_{ij}^{1}$*,*$c_{ij}^{2}$*,…,*$c_{ij}^{q}$, for which $c_{ij}^{q}=\frac{\partial y_{t+q}}{\partial \varepsilon_{t}}$, which means that during period *t*, when the perturbation term of the *j*th variable is increased by 1 unit, and the perturbation parameters of other periods remain constant, the influence of such changes on the value of the *i*th variable during period *t* + *q* can be quantified. Finally, by using Equation (2), the sum of the total influence of the *j*th perturbation term across all periods, $\varepsilon_{j}$, can be quantified.

The variance decomposition method is often used to perform in-depth studies of the dynamic characteristics of applied models. To determine the contribution of each perturbation term to the variance of *y_i_*, the relative variance contribution (RVC) rate is quantified, as follows [44]:

(3) $$RVC_{ji}^{\to }\left(s\right)=\frac{\sum_{q=0}^{s-1}{\left(c_{ij}^{0}\right)}^{2}\sigma_{jj}}{\sum_{j=1}^{k}\left\{\sum_{q=0}^{s-1}{\left(c_{ij}^{0}\right)}^{2}\sigma_{jj}\right\}}$$

In this formula, *ij* = 1, 2, *…*, *k*, and *σjj* represents the standard variation of the variable *j*th. If the value of $RVC_{ji\;}^{\to }\left(s\right)$ is large, it means that the influence of variable *j*th on variable *i*th is significant, and vice versa.

## 4. Results and Discussion

### 4.1. Unit Root Test

The establishment of a VAR model must ensure the stability of the variable sequence. To avoid the pseudo-regression phenomenon of the nonstationary sequence and to ensure the validity of the conclusion, a unit root test was first performed on the data (Table 1). The lnSI, lnCOD, and lnAN were tested, and none of the three variables was statistically significant, meaning they represent nonstationary sequences. After first-order difference processing, ΔlnSI, ΔlnCOD, and ΔlnAN all passed the test of statistical significance, and all three variables now represent stationary sequences. Therefore, all three variables are first-order single integral sequences, and the cointegration test can be further performed.

### 4.2. Cointegration Analysis

To further test whether cointegrating relationships existed among the variables, we applied the Johansen cointegration method. It can be seen from the test results (Table 2) that the values of the trace tests were all greater than the critical 5% confidence value, as was the largest eigenvalue, suggesting that there is at least one cointegration vector and that long-term stable cointegrating relationships exist among the variables.

### 4.3. Development of the VAR Models

Based on the ADF (augmented Dickey–Fuller) and cointegration tests, VAR models were established for the five northwestern provinces included in this study. According to the results of the five testing methods, namely LR (likelihood ratio test), FPE (final prediction error), AIC (Akaike information criterion), SC (Schwarz criterion), and HQ (Hannan–Quinn criterion), the lag coefficients of the VAR models were determined. Overall, the lag coefficient of the VAR models of the lnSI-lnCOD and lnSI-lnAN variable systems in Shaanxi, Gansu, and Qinghai was 2, or, in other words, VAR (2) was established. The lag coefficient of the VAR models of the lnSI-lnCOD and lnSI-lnAN variable systems in Ningxia and Xinjiang was 1, or, in other words, VAR (1) was established. Next, the root test was performed on the established models; the results showed that the reciprocals of all root modes were located within the unit circle and that the model had high stability (Table 3). Impulse response analysis and variance decomposition analysis were then conducted to ensure the validity of test outcomes.

### 4.4. Impulse Response Analysis and Variance Decomposition Analysis

According to the change in the impulse response curve, it becomes stable after around 10 years. Therefore, in the present study we set the lag interval as 0 to 10. The horizontal axis represents the response period, the vertical axis represents the response amplitude, and the solid line represents the degree of impulse response. According to the principle of variance decomposition, the mean square error (MSE) of pollutants discharged to the aquatic environment that are caused by the unit impact of the industrial structure change is decomposed into contributions of the variable of industrial structure adjustment, and the importance of this variable was further quantified to understand the degree to which the variables interact with each other.

**Figure 2:**
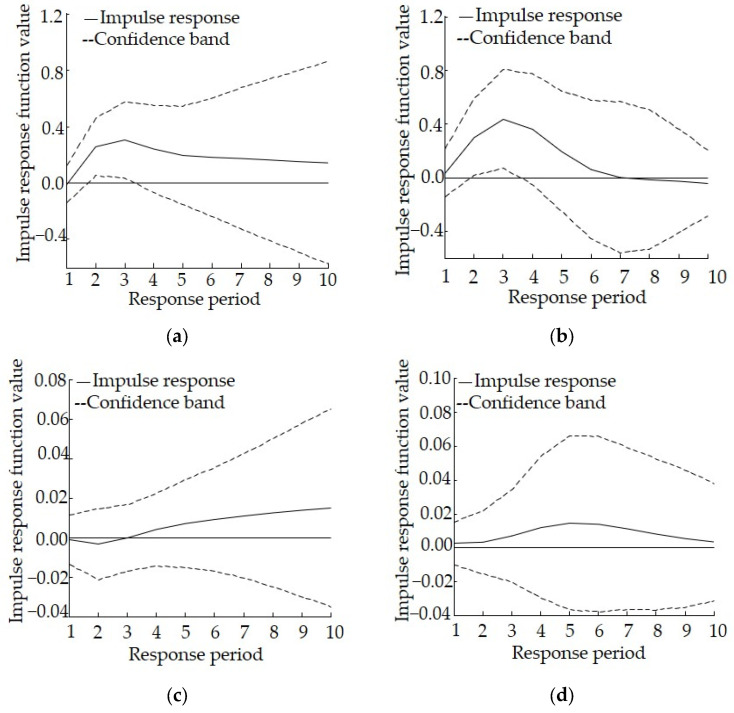
Impulse response relationship between industrial structure adjustment and the discharge of pollutants to the aquatic environment in Shaanxi, (**a**) Impulse response of lnCOD to lnSI, (**b**) Impulse response of lnAN to lnSI, (**c**) Impulse response of lnSI to lnCOD, (**d**) Impulse response of lnSI to lnAN.

The results of the impulse response analysis in Shaanxi are shown in Figure 2. As far as the impulse response of lnCOD to 1 unit of lnSI is concerned, it is both significant and persistent. The impulse response curve is always above the 0 axis, reaching a peak during the third period, then slowly falling back during the sixth period and becoming relatively stable, which suggests that industrial structure adjustment can effectively reduce COD. As far as the impulse response of lnSI to 1 unit of lnCOD is concerned, all values are positive and display an increasing trend except for during the first three periods, suggesting that COD discharge would impose a negative impact on industrial structure adjustment in the short term, although this effect could be reversed in the long term. As far as the impulse response of lnAN to 1 unit of lnSI is concerned, it shows some fluctuations. The response is rapid and strong during the first three periods, reaching a peak during the third period. Then, it decreases sharply and even shifts below the 0 axis after the eighth period, suggesting that industrial structure adjustment can effectively reduce the discharge of AN, although this reduction effect gradually weakens and even disappears during the late stages. As far as the impulse response of lnSI to 1 unit of lnAN is concerned, it fluctuates above 0, suggesting that the discharge of AN imposes a certain positive effect on industrial structure adjustment.

The results of the variance decomposition analysis for Shaanxi are shown in Table 4. The contribution rate of the discharge of COD to industrial structure gradually increases, although it generally remains low, with a value of 20.73% at the end of the period. The contribution rate of industrial structure to the discharge of COD reaches a maximum value in the fourth period and then falls back, but it generally remains high, with a value of 32.01% at the end of the period, suggesting that industrial structure plays a positive role in promoting the discharge of COD, industrial structure adjustment can effectively reduce the discharge of COD, and that there is a mutual promotion relationship between the two. The contribution rate of the discharge of AN to industrial structure gradually increases, although it generally remains low, with a value of 16.88% at the end of the period. In contrast, the overall contribution rate of industrial structure to the discharge of AN is high, with a value of 52.89% at the end of the period. Therefore, industrial structure plays a positive role in promoting the discharge of AN, while industrial structure adjustment can effectively reduce the discharge of AN. There is also a mutual promotion relationship between the two.

The response model shows that for Shaanxi, the discharge of COD would be affected for 10 years or more after the implementation of relevant policies related to industrial structure adjustment. Therefore, policies with the correct direction for industrial structure adjustment could ensure successful long-term effects. In addition, the adjustment of such policies comes at a low cost but with considerable environmental benefits. The difference lies in the fact that the impact related to the discharge of AN only occurs during the early stages, suggesting that it is very likely that relevant policies could have significant effects during the early implementation stage, but this effect might rapidly decrease or even become counterproductive. Therefore, before any counterproductive events occur, the timely revision and improvement of relevant policies will be necessary, depending on the actual social and economic development situation.

**Figure 3:**
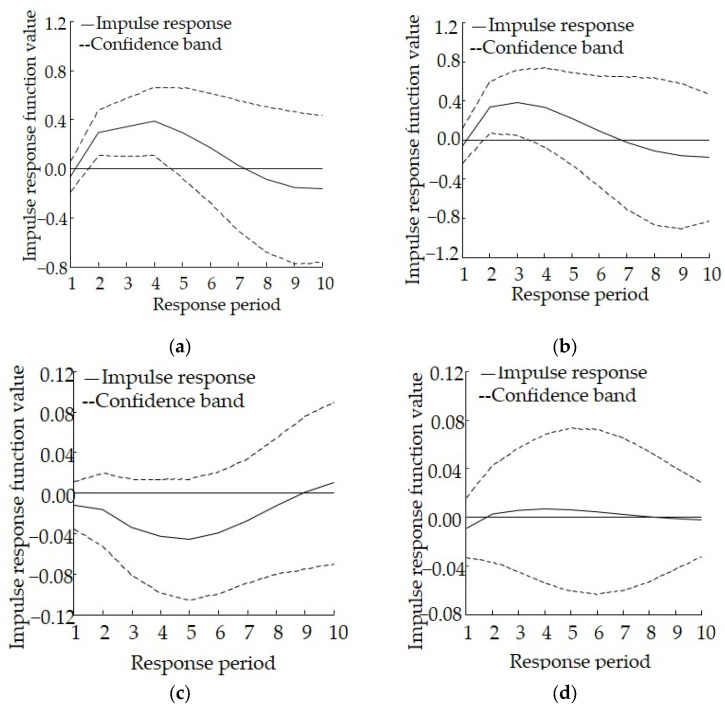
Impulse response relationship between industrial structure adjustment and the discharge of pollutants to the aquatic environment in Gansu, (**a**) Impulse response of lnCOD to lnSI, (**b**) Impulse response of lnAN to lnSI, (**c**) Impulse response of lnSI to lnCOD, (**d**) Impulse response of lnSI to lnAN.

The results of the impulse response analysis for Gansu are shown in Figure 3. Both the impulse response of lnCOD to 1 unit of lnSI and that of lnAN to 1 unit of lnSI share a very similar response path. Specifically, all values are negative during the first period and then begin to increase considerably during the second period, reaching the highest values in the third and fourth periods. They then begin to decline and decrease to negative values in the seventh to eighth periods, suggesting that industrial structure adjustment can reduce the discharge of COD and AN. However, this reduction effect gradually weakens and disappears during the later stages. As far as the impulse response of lnSI to 1 unit of lnCOD is concerned, all values are negative during the first period and then show a downward trend. The lowest value occurs during the fifth period and then the values begin to increase, becoming positive during the ninth period, suggesting that the discharge of COD imposes a negative effect on industrial structure adjustment. The difference lies in the fact that the impulse response of LnSI to lnAN is negative during the first period, turns positive in the second period, and then tends to coincide with the horizontal axis after the eighth period, suggesting that the discharge of AN has a relatively weak positive effect on industrial structure adjustment.

The results of the variance decomposition analysis for Gansu are shown in Table 5. The contribution rate of the discharge of COD and AN to industrial structure is 25.31% and 5.72%, respectively, at the end of the period, and the contribution rate of industrial structure to the discharge of COD and AN is 68.71% and 65.48%, respectively, at the end of the period. Overall, the impact of the latter is larger than that of the former, suggesting that industrial structure plays a positive role in promoting the discharge of COD and AN, whereas industrial structure adjustment can significantly reduce the discharge of COD and AN. In addition, there is a mutual promotion relationship between industrial structure and the discharge of COD, and between industrial structure and the discharge of AN.

The response model shows that for Gansu, relevant policies related to industrial structure adjustment could help reduce the discharge of COD and AN during the early stages, but the effect might gradually decrease in the medium term or even become counterproductive. Given this, special attention should be paid to the policy adjustment nodes, and periodic policy evaluation and revision are especially important.

**Figure 4:**
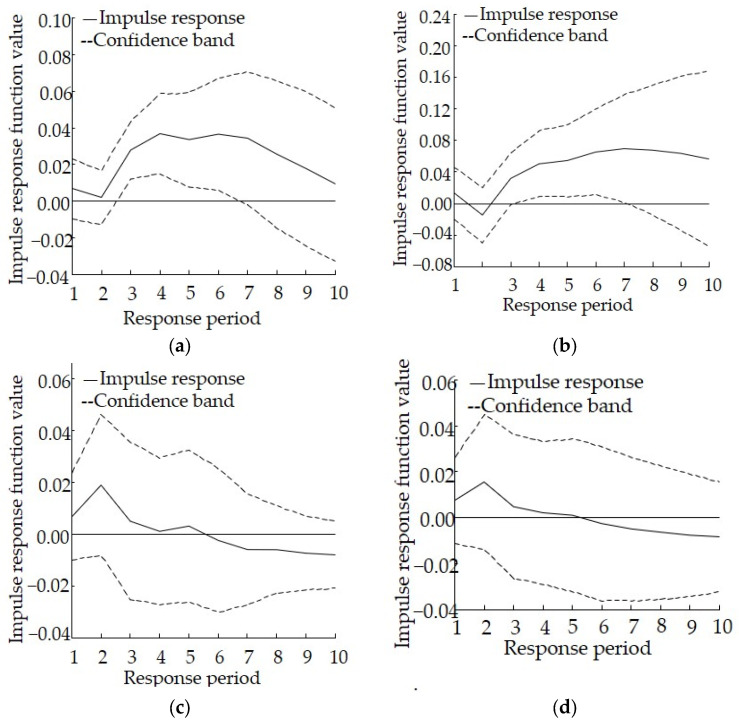
Impulse response relationship between industrial structure adjustment and the discharge of pollutants to the aquatic environment in Qinghai, (**a**) Impulse response of lnCOD to lnSI, (**b**) Impulse response of lnAN to lnSI, (**c**) Impulse response of lnSI to lnCOD, (**d**) Impulse response of lnSI to lnAN.

The results of the impulse response analysis in Qinghai are shown in Figure 4. As far as the impulse response of lnCOD to 1 unit of lnSI as well as lnAN to 1 unit of lnSI is concerned, they share a very similar response path. Specifically, all values are positive in the first period, then decrease to the lowest value during the second period. The main difference is that the response of lnAN to lnSI initially decreases to negative values and then remains positive in each period thereafter. In addition, it begins to decline gently again after the seventh period. Overall, industrial structure adjustment can help reduce the discharge of COD and AN. As far as the impulse response of lnSI to 1 unit of lnCOD as well as the impulse response of lnSI to 1 unit of lnAN is concerned, the response paths of the two are also similar. All values are positive in the first period and reach a maximum value during the second period. After that, all values begin to decline, with negative values after the fifth period. This suggests that the discharge of COD and AN imposes a positive effect on industrial structure adjustment in the early stages, but a negative effect in the later stages.

The results of the variance decomposition analysis for Qinghai are shown in Table 6. The contribution rate of the discharge of COD and AN to industrial structure is 5.05% and 5.43%, respectively, during the late stage, and the contribution rate of industrial structure to the discharge of COD and AN emission is 82.68% and 83.06%, respectively, during the late stage. Overall, the influence of the latter is greater than that of the former, suggesting that industrial structure plays an important role in promoting the discharge of COD and AN, whereas industrial structure adjustment can significantly reduce the discharge of COD and AN. In addition, certain mutual promotion relationships exist between industrial structure and the discharge of COD or AN.

The response model shows that for Qinghai, the effect is not obvious during the early stages of policy implementation, and industrial structure adjustment cannot effectively reduce the discharge of COD and AN. However, after 3 years, the effect gradually strengthens and becomes persistent. In other words, industrial structure adjustment can help reduce the discharge of COD and AN in a continuous and effective manner. Therefore, attention should be paid to developing appropriate management of the cycle of policy formulation, evaluation, revision, and improvement.

**Figure 5:**
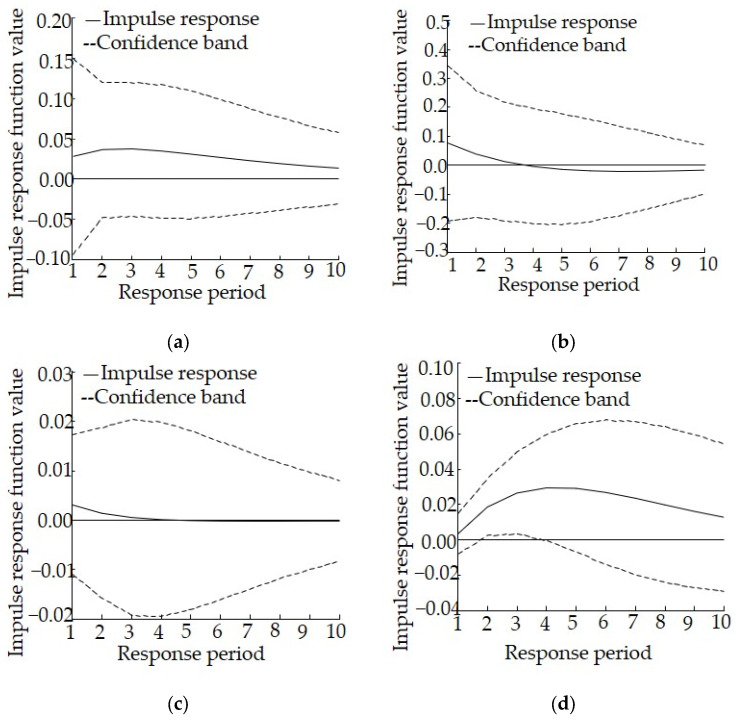
Impulse response relationship between industrial structure adjustment and the discharge of pollutants to the aquatic environment in Ningxia, (**a**) Impulse response of lnCOD to lnSI, (**b**) Impulse response of lnAN to lnSI, (**c**) Impulse response of lnSI to lnCOD, (**d**) Impulse response of lnSI to lnAN.

The results of the impulse response analysis in Ningxia are shown in Figure 5. As far as the impulse response of lnCOD to 1 unit of lnSI is concerned, the impulse response curve is always above the 0 axis, and the overall response path is relatively flat with small fluctuations, suggesting that industrial structure adjustment can help reduce the discharge of COD. As far as the impulse response of lnSI to 1 unit of lnCOD is concerned, the values during the first three periods are slightly positive, gradually decrease, and then essentially coincide with the 0 axis in the subsequent periods, suggesting that the discharge of COD imposes a weak positive effect on industrial structure adjustment during the early stages. As far as the impulse response of lnAN to 1 unit of lnSI is concerned, the values during the first four periods are positive and thereafter display a declining trend. All values become negative after the fourth period, suggesting that industrial structure adjustment can help reduce the discharge of AN in the early stages, but this reduction effect diminishes and rapidly disappears. As far as the impulse response of lnSI to 1 unit of lnAN is concerned, all values fluctuate above 0, suggesting that the discharge of AN imposes a certain positive effect on industrial structure adjustment.

The results of the variance decomposition analysis for Ningxia are shown in Table 7. The contribution rate of the discharge of COD to industrial structure and the contribution rate of industrial structure to the discharge of COD emissions is relatively small, suggesting a relatively weak relationship between industrial structure and the discharge of COD, with industrial structure adjustment playing only a minor role in reducing the discharge of COD, with insignificant effects. The contribution rate of the discharge of AN to industrial structure is 69.11% during the later stages, whereas the contribution rate of industrial structure to the discharge of AN is just 1.41% during the later stages. Clearly, the effect of the former is much greater than that of the latter, suggesting that industrial structure adjustment cannot significantly help to reduce the discharge of AN. In contrast, the discharge of AN plays a certain role in promoting industrial structure adjustment.

The response model shows that for Ningxia, the effect of policies related to industrial structure adjustment on the reduction of the discharge of COD and AN is insignificant. Therefore, we should not only focus on industrial structure adjustment but also fully mobilize all aspects of economic and social development, so that the goal of reducing pollutants entering the aquatic environment can be achieved through these joint efforts.

**Figure 6:**
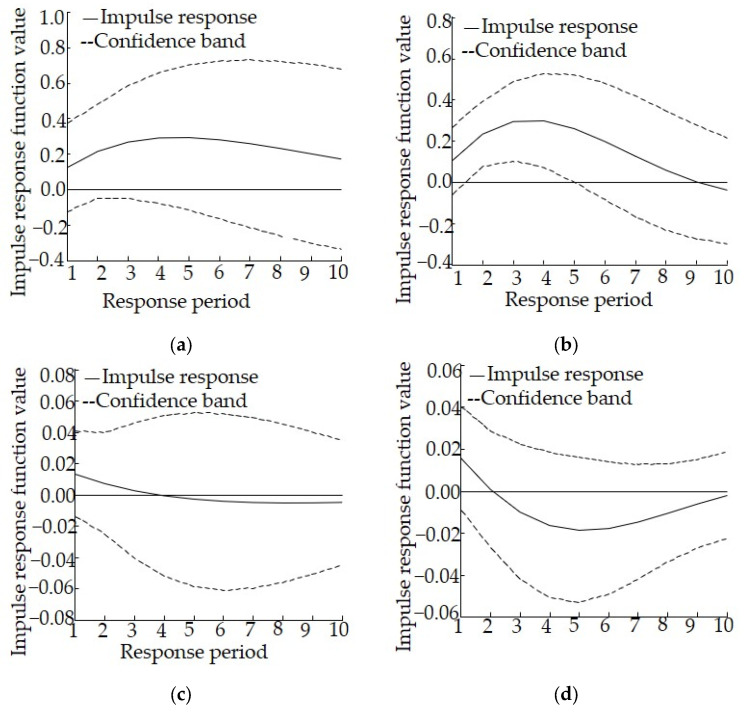
Impulse response relationship between industrial structure adjustment and the discharge of pollutants to the aquatic environment in Xinjiang, (**a**) Impulse response of lnCOD to lnSI, (**b**) Impulse response of lnAN to lnSI, (**c**) Impulse response of lnSI to lnCOD, (**d**) Impulse response of lnSI to lnAN.

The results of the impulse response analysis in Xinjiang are shown in Figure 6. As far as the impulse response of lnCOD to 1 unit of lnSI is concerned, the impulse response curve is always above the 0 axis, and the overall response path is relatively flat with small fluctuations, suggesting that industrial structure adjustment can help reduce the discharge of COD. As far as the impulse response of lnSI to 1 unit of lnCOD is concerned, the values during the first four periods are positive but gradually decrease, becoming negative during the subsequent periods. This suggests that the discharge of COD imposes a positive effect on industrial structure adjustment during the early stages, but this effect becomes negative during the middle and later stages. As far as the impulse response of lnAN to 1 unit of lnSI is concerned, the values during the first nine periods are all positive, and the overall response path is relatively flat. However, the values become negative after the ninth period, suggesting that industrial structure adjustment can help reduce the discharge of AN, but this reduction effect diminishes and disappears during the later stages. As far as the impulse response of lnSI to 1 unit of lnAN is concerned, all values become negative after the second period, suggesting that the discharge of AN imposes a certain negative effect on industrial structure adjustment.

The results of the variance decomposition analysis for Xinjiang are shown in Table 8. The contribution rate of the discharge of COD and AN to industrial structure is 4.74% and 23.57%, respectively, during the late stage, and the contribution rate of the industrial structure to the discharge of COD and AN emission is 45.85% and 60.94%, respectively, during the late stage. Overall, the influence of the latter is greater than that of the former, suggesting that industrial structure imposes a positive effect on the discharge of COD and AN, whereas industrial structure adjustment may help to significantly reduce the discharge of COD and AN. In addition, certain mutual promotion relationships exist between industrial structure and the discharge of COD or AN.

The response model shows that for Xinjiang, the discharge of COD would be affected for 10 years or more following the implementation of relevant policies related to industrial structure adjustment. Therefore, policies with the correct direction for industrial structure adjustment could ensure successful long-term effects. In addition, the adjustment of such policies comes at a low cost but with considerable environmental benefits. The difference is that the impact related to the discharge of AN cannot last until the later stages, suggesting that the effect of policies is rapidly reduced or even becomes counterproductive during the later stages of implementation. Therefore, special attention should be paid to the nodes of policy adjustment, and relevant policies should be revised and improved in a timely manner.

## 5. Conclusions

In general, industrial structure in most provinces in Northwest China imposes a certain positive effect over the discharge of pollutants to the aquatic environment. Adjusting industrial structure and reducing the proportion of secondary industry present can to some extent promote reductions in the discharge of pollutants to the aquatic environment. However, such beneficial effects may vary among different provinces.

Specifically, for Gansu, industrial structure adjustment could help reduce the discharge of pollutants to the aquatic environment effectively during the early stages, but this positive promotion effect gradually weakens and disappears during the later stages. In Qinghai, industrial structure adjustment cannot help reduce the discharge of pollutants to the aquatic environment effectively during the early stages, but a positive promotion effect gradually increases and continues to function later. The performance in Shaanxi and Xinjiang was quite similar, with industrial structure adjustment helping to effectively reduce the discharge of pollutants to the aquatic environment over a long period of time. This positive effect can play a more sustained and stable role. For Ningxia, industrial structure adjustment can not only help significantly reduce the discharge of pollutants to the aquatic environment but also displays a significant positive effect.

Given the specific conditions and characteristics of the region under study, relevant policies for industrial structure adjustment should be formulated and implemented. For Shaanxi and Xinjiang, policies with the correct direction for industrial structure adjustment should be maintained to ensure successful long-term effects. The adjustment of such policies comes at a low cost but with considerable environmental benefits, although it is still necessary to pay attention to the policy adjustment nodes and to revise and improve relevant policies in a timely manner. For Gansu and Qinghai, special attention should be paid to the policy adjustment nodes, and the cycle of policy formulation, evaluation, revision, and improvement should be well managed, with particular emphasis on periodic policy evaluation and revision. For Ningxia, we should not only focus on industrial structure adjustment but also fully mobilize multiple facets of economic and social development, so that the aim of reducing the discharge of pollutants to the aquatic environment can be realized.

This work had some limitations. It is very useful to identify particular chemical classes of pollutant and to support control measures, and prioritization schemes should be established for those compounds that cause the most harm to the environment. However, these aspects were not considered in this research, as the relevant data were not available. This should be investigated in future research. Additionally, alternative methods, such as principal component analysis (PCA) and hierarchical clustering analysis (HCA), could be employed in future research, to reveal further details from different perspectives.

## Figures and Tables

**Table 1:** Results of the unit root test.

Province	Variable	Test Type	ADF Value	Confidence Value			Conclusion	*p*-Value
		(C,T,P)		1%	5%	10%		
Shaanxi	LnSI	(C,T,0)	−0.692	−4.571	−3.690	−3.286	nonstationary	0.957
	ΔLnSI	(C,T,0)	−3.328	−4.616	−3.710	−3.297	stationary	0.095
	LnCOD	(C,T,0)	0.574	−3.857	−3.040	−2.660	nonstationary	0.984
	ΔLnCOD	(C,T,0)	−3.309	−3.886	−3.052	−2.666	stationary	0.030
	LnAN	(C,T,0)	−0.055	−4.571	−3.690	−3.286	nonstationary	0.991
	ΔLnAN	(C,T,0)	−3.412	−4.616	−3.7104	−3.297	stationary	0.082
Gansu	LnSI	(C,T,0)	−0.275	−4.571	−3.690	−3.286	nonstationary	0.984
	ΔLnSI	(C,T,0)	−3.655	−4.616	−3.710	−3.297	stationary	0.055
	LnCOD	(C,T,0)	−0.876	−4.571	−3.690	−3.286	nonstationary	0.936
	ΔLnCOD	(C,T,0)	−3.821	−4.616	−3.710	−3.297	stationary	0.041
	LnAN	(C,0,0)	−0.808	−3.857	−3.040	−2.660	nonstationary	0.792
	ΔLnAN	(C,0,0)	−4.457	−3.886	−3.052	−2.666	stationary	0.003
Qinghai	LnSI	(C,T,0)	0.480	−4.571	−3.690	−3.286	nonstationary	0.998
	ΔLnSI	(C,T,0)	−3.983	−4.616	−3.710	−3.297	stationary	0.031
	LnCOD	(C,T,0)	−0.871	−4.571	−3.690	−3.286	nonstationary	0.937
	ΔLnCOD	(C,T,0)	−4.843	−4.616	−3.710	−3.297	stationary	0.006
	LnAN	(C,T,0)	−1.366	−4.571	−3.690	−3.286	nonstationary	0.835
	ΔLnAN	(C,T,0)	−6.275	−4.616	−3.710	−3.297	stationary	0.001
Ningxia	LnSI	(C,T,0)	−0.020	−4.571	−3.690	−3.286	nonstationary	0.992
	ΔLnSI	(C,T,0)	−4.586	−4.616	−3.710	−3.297	stationary	0.010
	LnCOD	(C,0,0)	−2.431	−3.857	−3.040	−2.660	nonstationary	0.147
	ΔLnCOD	(C,0,0)	−4.898	−3.886	−3.052	−2.666	stationary	0.001
	LnAN	(C,T,0)	−1.590	−4.571	−3.690	−3.286	nonstationary	0.755
	ΔLnAN	(C,T,0)	−5.121	−4.616	−3.710	−3.297	stationary	0.004
Xinjiang	LnSI	(C,0,0)	−1.324	−3.857	−3.040	−2.660	nonstationary	0.594
	ΔLnSI	(C,0,0)	−3.580	−3.886	−3.052	−2.666	stationary	0.018
	LnCOD	(C,T,0)	−0.903	−4.571	−3.690	−3.286	nonstationary	0.933
	ΔLnCOD	(C,T,0)	−4.145	−4.616	−3.710	−3.297	stationary	0.023
	LnAN	(C,T,0)	−0.119	−4.571	−3.690	−3.286	nonstationary	0.989
	ΔLnAN	(C,T,0)	−3.415	−4.616	−3.710	−3.297	stationary	0.082

**Table 2:** Test results of the Johansen cointegration analysis.

Province		Trace				Maximum Eigenvalue Test			
		Number of Cointegration Equations	Root Value	Trace Value	5% Confidence Value	Number of Cointegration Equations	Root Value	Trace Value	5% Confidence Value
		Rank ≤ (r)				Rank ≤ (r)			
Shaanxi	lnSI-lnCOD	r = 0	0.721	32.8	25.872	r = 0	0.721	20.431	19.387
		r ≤ 1	0.538	12.369	12.518	r = 1	0.538	12.369	12.518
	lnSI-lnAN	r = 0	0.783	30.304	25.872	r = 0	0.783	22.93	19.387
		r ≤ 1	0.388	7.374	12.518	r = 1	0.388	7.374	12.518
Gansu	lnSI-lnCOD	r = 0	0.935	47.099	25.872	r = 0	0.935	40.994	19.387
		r ≤ 1	0.334	6.105	12.518	r = 1	0.334	6.105	12.518
	lnSI-lnAN	r = 0	0.773	27.878	25.872	r = 0	0.773	22.264	19.387
		r ≤ 1	0.312	5.614	12.518	r = 1	0.312	5.614	12.518
Qinghai	lnSI-lnCOD	r = 0	0.716	26.894	25.872	r = 0	0.716	20.148	19.387
		r ≤ 1	0.344	6.746	12.518	r = 1	0.344	6.746	12.518
	lnSI-lnAN	r = 0	0.745	27.977	25.872	r = 0	0.745	20.482	19.387
		r ≤ 1	0.393	7.496	12.518	r = 1	0.393	7.496	12.518
Ningxia	lnSI-lnCOD	r = 0	0.841	45.316	25.872	r = 0	0.841	29.42	19.387
		r ≤ 1	0.63	15.895	12.518	r = 1	0.63	15.895	12.518
	lnSI-lnAN	r = 0	0.77	28.978	25.872	r = 0	0.77	23.513	19.387
		r ≤ 1	0.289	5.465	12.518	r = 1	0.289	5.465	12.518
Xinjiang	lnSI-lnCOD	r = 0	0.628	25.312	15.495	r = 0	0.628	14.849	14.265
		r ≤ 1	0.502	10.463	3.841	r = 1	0.502	10.463	3.841
	lnSI-lnAN	r = 0	0.845	34.887	25.872	r = 0	0.845	28.005	19.387
		r ≤ 1	0.368	6.882	12.518	r = 1	0.368	6.882	12.518

**Table 3:** The optimal lag period of the VAR models.

Province		Lag	LR	FPE	AIC	SC	HQ
Shaanxi	lnSI-lnCOD	0	NA	0.002	−0.25	−0.15	−0.24
		1	47.73	0.0001	−3.19	−2.9	−3.16
		2	12.67 *	8.09 *	−3.78 *	−3.29 *	−3.73 *
	lnSI-lnAN	0	NA	0.001	−0.84	−0.75	−0.83
		1	32.26	0.0002	−2.68	−2.38	−2.65
		2	11.52 *	0.0001 *	−3.16 *	−2.67 *	−3.11 *
Gansu	lnSI-lnCOD	0	NA	0.002	−0.21	−0.11	−0.2
		1	37.97 *	0.0002	−2.45	−2.15 *	−2.42
		2	7.66	0.0002 *	−2.615 *	−2.13	−2.567 *
	lnSI-lnAN	0	NA	0.004	0.16	0.26	0.17
		1	29.80 *	0.0007	−1.5	−1.2	−1.47
		2	9.29	0.0005 *	−1.80 *	−1.31 *	−1.75 *
Qinghai	lnSI-lnCOD	0	NA	4.69	−4.29	−4.19	−4.28
		1	40.14	4.3	−6.69	−6.4	−6.66
		2	13.75 *	2.25 *	−7.36 *	−6.87 *	−7.31 *
	lnSI-lnAN	0	NA	0.0002	−2.53	−2.44	−2.52
		1	45.61	1.69	−5.32	−5.03	−5.29
		2	10.64 *	1.14 *	−5.73 *	−5.24 *	−5.68 *
Ningxia	lnSI-lnCOD	0	NA	0.0001	−2.89	−2.8	−2.88
		1	26.34 *	4.66 *	−4.30 *	−4.01 *	−4.27 *
		2	3.46	5.75	−4.12	−3.63	−4.07
	lnSI-lnAN	0	NA	0.0018	−0.6	−0.5	−0.59
		1	36.36 *	0.0002 *	−2.72 *	−2.43 *	−2.69 *
		2	3.28	2.83	−2.53	−2.04	−2.48
Xinjiang	lnSI-lnCOD	0	NA	0.01	0.52	0.62	0.53
		1	26.22 *	0.001 *	−0.87 *	−0.58 *	−0.85 *
		2	1.85	2.02	−0.56	−0.07	−0.51
	lnSI-lnAN	0	NA	0.003	0.11	0.21	0.12
		1	5.93 *	0.0004 *	−1.98 *	−1.69 *	−1.95 *
		2	1.28	7.02	−1.62	−1.13	−1.57

Note: The optimal lag order selected according to the corresponding criteria is marked by *.

**Table 4:** Forecast variance decomposition of the discharge of pollutants to the aquatic environment in response to industrial structure adjustment in Shaanxi.

Response Period	Variance Decomposition of lnSI		Variance Decomposition of lnCOD		Variance Decomposition of lnSI		Variance Decomposition of lnAN	
	lnSI	lnCOD	lnSI	lnCOD	lnSI	lnAN	lnSI	lnAN
1	100	0	0.12	99.88	100	0	1	99
2	99.73	0.27	32.82	67.18	100	0	24.96	75.04
3	99.77	0.23	46.37	53.63	99.14	0.86	45.26	54.74
4	98.84	1.16	47.28	52.72	95.54	4.46	53.23	46.77
5	96.8	3.2	44.63	55.37	90.57	9.43	54.54	45.46
6	94.07	5.93	41.65	58.35	86.85	13.15	53.87	46.13
7	90.82	9.18	38.95	61.05	84.84	15.16	53.15	46.85
8	87.16	12.84	36.46	63.54	83.85	16.15	52.84	47.16
9	83.26	16.74	34.13	65.87	83.35	16.65	52.81	47.19
10	79.27	20.73	32.01	67.99	83.12	16.88	52.89	47.11

**Table 5:** Forecast variance decomposition of the discharge of pollutants to the aquatic environment in response to industrial structure adjustment in Gansu.

Response Period	Variance Decomposition of lnSI		Variance Decomposition of lnCOD		Variance Decomposition of lnSI		Variance Decomposition of lnAN	
	lnSI	lnCOD	lnSI	lnCOD	lnSI	lnAN	lnSI	lnAN
1	100	0	6.3	93.7	100	0	2.99	97.01
2	99.94	0.06	51.08	48.92	97.1	2.9	37.93	62.07
3	96.96	3.04	68.94	31.06	95.62	4.38	53.25	46.75
4	91.47	8.53	78.65	21.35	94.84	5.16	59.86	40.14
5	84.24	15.76	78.58	21.42	94.48	5.52	62.04	37.96
6	77.9	22.1	74.01	25.99	94.34	5.66	62.36	37.64
7	74.28	25.72	69.35	30.65	94.31	5.69	62.4	37.6
8	73.56	26.44	67.19	32.81	94.32	5.68	63.02	36.98
9	74.22	25.78	67.58	32.42	94.31	5.69	64.2	35.8
10	74.69	25.31	68.71	31.29	94.28	5.72	65.48	34.52

**Table 6:** Forecast variance decomposition of the discharge of pollutants to the aquatic environment in response to industrial structure adjustment in Qinghai.

Response Period	Variance Decomposition of lnSI		Variance Decomposition of lnCOD		Variance Decomposition of lnSI		Variance Decomposition of lnAN	
	lnSI	lnCOD	lnSI	lnCOD	lnSI	lnAN	lnSI	lnAN
1	100	0	3.9	96.1	100	0	3.73	96.27
2	96.65	3.35	4.15	95.85	99	1	7.93	92.07
3	97.62	2.38	42.19	57.81	99.05	0.95	23.04	76.96
4	97.44	2.56	64.78	35.22	98.6	1.4	44.75	55.25
5	97.64	2.36	73.52	26.48	98.2	1.8	58.7	41.3
6	97.13	2.87	78.86	21.14	97.48	2.52	69.58	30.42
7	96.27	3.73	82.11	17.89	96.69	3.31	76.37	23.63
8	95.69	4.31	83.08	16.92	95.93	4.07	80.08	19.92
9	95.22	4.78	82.97	17.03	95.21	4.79	82.08	17.92
10	94.95	5.05	82.68	17.32	94.57	5.43	83.06	16.94

**Table 7:** Forecast variance decomposition of the discharge of pollutants to the aquatic environment in response to industrial structure adjustment in Ningxia.

Response Period	Variance Decomposition of lnSI		Variance Decomposition of lnCOD		Variance Decomposition of lnSI		Variance Decomposition of lnAN	
	lnSI	lnCOD	lnSI	lnCOD	lnSI	lnAN	lnSI	lnAN
1	100	0	1.14	98.86	100	0	1.74	98.26
2	99.91	0.09	2.4	97.6	80.82	19.18	1.43	98.57
3	99.79	0.21	3.73	96.27	62.35	37.65	1.25	98.75
4	99.69	0.31	4.88	95.12	50.18	49.82	1.17	98.83
5	99.62	0.38	5.79	94.21	42.57	57.43	1.17	98.83
6	99.56	0.44	6.46	93.54	37.77	62.23	1.21	98.79
7	99.52	0.48	6.95	93.05	34.74	65.26	1.27	98.73
8	99.5	0.5	7.29	92.71	32.81	67.19	1.32	98.68
9	99.48	0.52	7.52	92.48	31.62	68.38	1.37	98.63
10	99.47	0.53	7.69	92.31	30.89	69.11	1.41	98.59

**Table 8:** Forecast variance decomposition of the discharge of pollutants to the aquatic environment in response to industrial structure adjustment in Xinjiang.

Response Period	Variance Decomposition of lnSI		Variance Decomposition of lnCOD		Variance Decomposition of lnSI		Variance Decomposition of lnAN	
	lnSI	lnCOD	lnSI	lnCOD	lnSI	lnAN	lnSI	lnAN
1	100	0	5.39	94.61	100	0	8.57	91.43
2	99.73	0.27	12.13	87.87	96.34	3.66	30.07	69.93
3	99.21	0.79	19.37	80.63	90.7	9.3	49.36	50.64
4	98.55	1.45	26.11	73.89	84.99	15.01	60.53	39.47
5	97.85	2.15	31.84	68.16	80.36	19.64	64.83	35.17
6	97.16	2.84	36.45	63.55	77.36	22.64	65.15	34.85
7	96.54	3.46	40	60	75.99	24.01	63.81	36.19
8	96.01	3.99	42.64	57.36	75.79	24.21	62.27	37.73
9	95.59	4.41	44.53	55.47	76.1	23.9	61.26	38.74
10	95.26	4.74	45.85	54.15	76.43	23.57	60.94	39.06

## Data Availability

The data used to support the findings of this study are available from the corresponding author upon request.
